# Compassion Fatigue: An Application of the Concept to Informal Caregivers of Family Members with Dementia

**DOI:** 10.1155/2011/408024

**Published:** 2011-09-08

**Authors:** Jennifer R. Day, Ruth A. Anderson

**Affiliations:** Duke University School of Nursing, 307 Trent Drive, DUMC 3322, Durham, NC 27710, USA

## Abstract

*Introduction*. Compassion fatigue is a concept used with increasing frequency in the nursing literature. The objective of this paper is to identify common themes across the literature and to apply these themes, and an existing model of compassion fatigue, to informal caregivers for family members with dementia. *Findings*. Caregivers for family members with dementia may be at risk for developing compassion fatigue. The model of compassion fatigue provides an informative framework for understanding compassion fatigue in the informal caregiver population. Limitations of the model when applied to this population were identified as traumatic memories and the emotional relationship between parent and child, suggesting areas for future research. *Conclusions*. Research is needed to better understand the impact of compassion fatigue on informal caregivers through qualitative interviews, to identify informal caregivers at risk for compassion fatigue, and to provide an empirical basis for developing nursing interventions for caregivers experiencing compassion fatigue.

## 1. Introduction

Compassion fatigue was introduced to the health care community as a unique form of burnout experienced by those in caring professions, particularly palliative care and oncology nurses [1]. Later, other health care professions such as social work, medicine, and psychology adopted the concept [2–5]. Many definitions of compassion fatigue have been offered by researchers and authors, the most common being that compassion fatigue is an adverse consequence of caring for individuals in need and the caregiver may experience the symptoms of anger, depression, and apathy [1, 6, 7]. Family caregivers, particularly those caring for older adults with dementia, display many of the characteristics of compassion fatigue. Caregivers for family members with dementia experience depression, anxiety, and stress [8–15] and also feelings of resentment, helplessness, and hopelessness, in addition to feeling that they have little free time [16]. Caregivers who have these feelings and experiences on top of the emotionally laden filial caregiving relationship may be suffering from compassion fatigue, yet compassion fatigue has not been explored for informal caregivers of family members with dementia. 

Informal caregivers for family members with dementia who develop compassion fatigue may terminate the caregiving relationship though premature nursing home admission or relinquishing care to another family member, and there may also be increased risk for abuse or neglect [13, 14]. Supporting these caregivers may improve outcomes for people with dementia because individuals who are cared for at home have lower morbidity and mortality than those in institutional settings [17], and caring for family members at home provides a significant decrease in cost to society [17, 18]. In order for the professional health care community to support these caregivers, compassion fatigue must be fully explored in this population of caregivers. Therefore, the objective of this paper is to identify common themes across the literature on compassion fatigue and to apply these themes and the existing model of compassion fatigue to informal caregivers for family members with dementia.

## 2. Compassion Fatigue: Themes from the Literature

Historically, the health care literature has not presented a consistent definition of compassion fatigue. Compassion fatigue, a term introduced by Joinson [1] and developed by Figley [5], has been defined interchangeably with secondary traumatic stress and secondary traumatic stress disorder, vicarious traumatization, and burnout [2, 3, 7, 19], thus creating confusion regarding its definition. For example, authors of a 2008 literature review of compassion fatigue as experienced by cancer-care providers stated that they were unable to generate a definition based on the 14 studies reviewed [20].  Table 1 contains the unique definitions of compassion fatigue found in earlier literature from nursing, and all articles by Figley, or authors adapting his definition.

Several themes arise from the definitions in the literature of compassion fatigue. Compassion fatigue (a) is dependent on a caring relationship between the caregiver and a care recipient, who is suffering or traumatized [1, 2, 7, 19, 21, 22], (b) is a form of burnout [1, 7], (c) has an acute onset [2, 4], and (d) has negative emotional responses for the caregiver such as helplessness, hopelessness, isolation, and apathy or an inability to be empathic [1, 3, 6, 19]. These four themes and Figley's model of the compassion fatigue process [2] provide a basis for exploring whether compassion fatigue is a concept that applies to informal caregivers for family members with dementia.

## 3. Compassion Fatigue: Applied to Informal Caregivers

**Figure 1:**
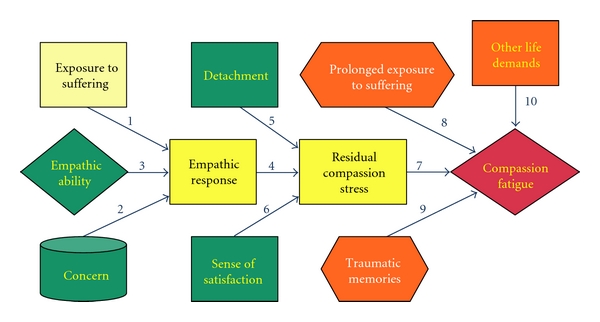
Compassion fatigue process [23].

As shown in Figure 1 [23], compassion fatigue is a process. According to Figley's model, the caregiver must have concern and an empathic ability or feel motivated to respond when they perceive that the care recipient is suffering [2]. When caregivers have this empathic response, coupled with an unwillingness or inability to detach from the caregiving situation and the absence of feelings of satisfaction, the caregiver develops compassion stress [2]. Compassion stress results in compassion fatigue if the caregiver has prolonged exposure to suffering coupled with traumatic memories and competing life demands [2]. 

Compassion fatigue has not been specifically studied in informal caregivers, but many of the characteristics of compassion fatigue are recognizable in informal caregivers, particularly those caring for family members with dementia. Characteristics of compassion fatigue displayed in formal caregivers include, but are not limited to, apathy, depression, and anxiety [1, 2, 22]. Dementia caregivers experience stress and have also been found to experience anxiety and depression [8–10, 12, 13]. It is likely, therefore, that informal caregivers are at risk for compassion fatigue.

To analyze the applicability of the concept to informal caregivers, we apply Figley's model of the compassion fatigue process (Figure 1) [23] to informal caregivers, explore the possible differences in compassion fatigue between formal and informal caregivers, and use the results of the analysis to suggest future directions for research on compassion fatigue in family caregivers.

### 3.1. Empathic Ability, Concern, and Attachment

Although compassion fatigue has not been specifically studied in family caregivers for a family member with dementia, their experiences appear to place them at risk for developing compassion fatigue. For example, a loving emotional relationship between care recipient and caregiver is important in the family caregiving dyad and we propose this places the caregiver at particular risk for compassion fatigue. The caregiver-care recipient relationship provides a source for the caregiver's desire to respond to suffering and the caregiver's ability to recognize their family member's suffering. The following quote from a daughter about her relationship with her mother demonstrates an affectionate relationship: “If I didn't love my mother unconditionally, this experience wouldn't hurt so deeply. So I am grateful to have loved truly and deeply and to suffer now, than never to have had such an amazing relationship in the first place” [24]. Although this daughter does not display compassion fatigue, she may be at risk given her close relationship with her mother. 

Premorbid relationship quality impacts the caregiving relationship [25]. Research shows that caregivers who had a positive relationship have less strain during caregiving and are able to find meaning in caregiving [26–28]. Compassion is attributable to the quality of the relationship between two people [29]. Compassion happens in the moment and is not about how a person is, but what a person does; “It is not something I have, and give to you. It manifests in the relationship between beings” [29]. The relationship quality is likely central to the reason the caregiver experiences compassion fatigue and provides for the emotional attachment the caregiver has to the care recipient, thus making informal caregivers particularly vulnerable to compassion fatigue. Relationships with an emotional attachment may affect the way the caregiver perceives suffering by the care recipient and influence the amount of empathy that the caregiver feels.

Informal caregivers for family members with dementia may be at an even greater risk for compassion fatigue than formal care providers given the emotional attachment to the care recipient. In the compassion fatigue process, detachment is the ability of the provider to distance themselves from the suffering [2]. The strong emotional attachment between parent and child may prohibit the family caregiver from detaching and therefore removes an essential coping mechanism utilized by formal care providers [2, 30]. Thus, emotional attachment is an important aspect for future studies about informal caregivers and compassion fatigue.

### 3.2. Exposure to Perceived Suffering

Studies suggest that caregivers feel that their family member with dementia is suffering, regardless of how much the person with dementia feels himself or herself to be suffering [22, 31–33]. Schulz et al. [22] found that higher caregiver compassion was related to family member suffering but stated that the worst case occurs when “suffering is chronic and intense with low perceived ability to affect its course” [22]. Chronic suffering is particularly true for dementia patients and many family members are helpless to change the trajectory and thus are at greater risk to develop compassion fatigue. Dementia caregivers care for longer periods of time than caregivers for other chronic illnesses [11]; 71% care for more than a year and 32% care for five years or more [34]. Caring for a parent with dementia was described by one adult child as “the funeral that never ends” [35] and demonstrates a prolonged exposure for the caregivers to their family members' suffering. 

In addition, a qualitative, secondary analysis of interviews with nurse-daughters caring for elderly parents identified an association between parental suffering and compassion fatigue [36]. One daughter described the difference between caring for a parent versus caring for a nonfamily member this way, “When I saw my parents suffering, I suffered… When it's your parent and someone you love so intensely, you just want more for them to be safe and healthy” [36]. There is a clear relationship between the affection or emotional attachment to the parent and the daughter's perception of suffering [36].

Whereas formal care providers may be able to take time away from people who are suffering [1–3, 7, 21], family caregivers may not have this opportunity [22, 37]. This may place family caregivers at a greater risk for compassion fatigue than formal caregivers. In addition, family caregivers are often caring for long periods of time, around-the-clock, and thus this constant exposure to suffering is especially prolonged for informal caregivers for family members with dementia.

### 3.3. Sense of Satisfaction

Feelings of fulfillment and contentment from caring for a family member with dementia may protect family caregivers from compassion fatigue. Without a sense of satisfaction in caring, however, the caregiver is at risk for compassion fatigue. Dementia caregivers are capable of experiencing satisfaction, but their satisfaction results from the family member with dementia's well-being, as well as from receiving appreciation for the care they are providing [38], both of which are compromised by the dementing condition. There are also racial differences with caregiving satisfaction. A large, multisite study with 720 participants comparing Caucasian and African American family caregivers found that Caucasian caregivers had a lower perceived benefit from caregiving than the African American caregivers when controlling for socioeconomic status, gender, relationship, and age [39]. Caucasian caregivers also demonstrated a decrease in life satisfaction over time, whereas the African American caregivers had a continued high level of life satisfaction [39]. Thus, the race or ethnicity of a caregiver may place him or her at greater risk for compassion fatigue.

### 3.4. Traumatic Memories

We propose that the informal caregiver may develop compassion fatigue without traumatic memories, and this is where application of Figley's model to informal caregivers may diverge from formal healthcare providers. In reviewing the history of the concept of compassion fatigue, the definition has changed from a definition without a focus on traumatic memories [1] to one that incorporates these elements [2]. Compassion fatigue, according to Joinson, is caused by unavoidable external sources, is difficult to recognize, and leads to a caregiver becoming “angry, ineffective, apathetic, and depressed” [1]. Compassion fatigue may be caused when a nurse encounters more stress than he or she is able to cope with and the nurse's ability to function is compromised [1]. Joinson's definition does not include traumatic memories; this element was added to the definition by Figley when he represented compassion fatigue as a “state of tension and preoccupation with the traumatized patients by re-experiencing the traumatic events, avoidance/numbing of reminders [sic] persistent arousal (e.g., anxiety) associated with the patient” [2]. We propose that some definitions of compassion fatigue exclude the caregiver's own personal traumatic memories and compassion fatigue is therefore applicable to informal caregivers, whether or not they have traumatic memories. Future informal caregiving research may illicit traumatic memories from family caregivers and will allow for the exploration of possible traumatic memories related to caregiving in this population.

### 3.5. Life Demands

The time caregivers spend on caregiving removes them from other relationships. Informal caregivers for family members with dementia often find themselves alone during the day with their family member, and, while this relationship is valuable, it does not replace peer relationships. Caregivers become isolated and often feel there is no one they can talk to about their feelings and that friends cannot relate to them [40]. Hirschfeld's [16] survey of 30 caregivers found that caregivers felt resentful, helpless, and hopeless and that they had little free time. When a caregiver has little free time, they are unable to participate in activities focusing on themselves and fostering other relationships.

It is likely that multiple life demands can also contribute to a caregiver developing compassion fatigue. Dementia caregivers are frequently faced with situations of burden and the more dependent the care recipient, the higher the burden and demands. Layering on the emotional attachment of the family caregiving situation may facilitate development of compassion fatigue in ways that are yet unknown, and likely unique to family caregivers. The life demands of a family caregiver are different from formal caregivers [16, 40], and we propose that these demands may make it more difficult for family caregivers to avoid developing compassion fatigue. Family caregivers appear to have fewer outlets for support and buffers against the compassion fatigue process than do formal caregivers.

## 4. Indicators of Compassion Fatigue

Our analysis of the literature presenting definitions of compassion fatigue suggests that the indications of compassion fatigue present in formal healthcare providers, such as hopelessness, helplessness, emotional disengagement, and apathy, also are present in caregivers for older adults with dementia. Compassion fatigue is a combination of these factors, not each independently, and may present differently in informal caregivers than it does in formal healthcare providers. The literature on potential indicators of compassion fatigue is presented to help establish whether further research in this area would be fruitful.

### 4.1. Hopelessness

Family caregivers, particularly caregivers for older adults with dementia, exhibit hopelessness. Hope is defined as “a feeling of desire for something, usually with confidence in the possibility of its fulfillment” [41]. Caregivers who are experiencing hopelessness will feel the impossibility of a desire to be fulfilled. A study of 129 caregivers and 145 noncaregivers in Brazil compared levels of depression, anxiety, hopelessness, and stress [42]. Caregivers had significant differences from noncaregivers on the hopelessness measure, with higher levels of hopelessness [42]. Hopelessness was also associated with higher levels of anxiety and depression in the caregivers [42]. In this study, the relationship between caregiver and the person with dementia and stage of dementia were not related to level of hopelessness [42].

### 4.2. Helplessness

Caregivers who feel helpless may also feel powerless in the caregiving situation. Helplessness in caregivers for family members with dementia is associated with the frequency of depressive behaviors in the care recipient, the caregiver's appraisal of these depressive behaviors, and the caregiver's appraisal of disruptive behaviors [43]. Caregivers are more likely to feel helpless when they do not understand a family member's depression [43]. Male caregivers and Christian caregivers were more likely to have feelings of helplessness compared to female caregivers and Muslim or Druze caregivers [43]. Caregivers of family members with cancer describe helplessness and powerlessness, particularly related to the perception of suffering [44]. Participants described suffering not only as pain but as an existential suffering, including impaired function in daily life, reduced autonomy, and loneliness [44]. Helplessness and powerlessness were related to the caregiver's perception of the patient's fading away and feelings of insufficiency [44], and dementia caregivers also report feeling that their family member is fading away [22].

### 4.3. Apathy

Apathy is a lack of interest or concern and has been included in the definition of compassion fatigue in professional caregivers [1]. No studies were found that described caregivers experiencing apathy in relationship to caring for family members with dementia. Because there is no research on the concept of compassion fatigue in family caregivers, the meaning of apathy for this population is yet to be understood.

### 4.4. Emotional Disengagement or Isolation

Caregivers with compassion fatigue may have emotional disengagement or active withdrawal from and avoidance of the caregiving situation. Emotional disengagement has been explored in spouses of patients undergoing cardiac rehabilitation [45]. O'Farrell et al. [45] found that spouses who were distressed coped using disengagement strategies significantly more than nondistressed spouses. Qualitative interviews with 10 family caregivers about stigma, particularly family stigma that comes from being associated with a relative with a stigmatic mark [46], revealed that children caregivers who had negative emotions to caregiving, such as embarrassment, shame, and particularly disgust, reported a decrease in involvement. One caregiver stated, “I come to visit her and I see her…like a small bird…toothless…all wrinkled. I approach her but I cannot talk to her, I cannot hug her, kiss her” [47]. Compassion fatigue, like the family stigma that causes the family members of a person with dementia to have feelings of disgust, embarrassment, and decreased involvement, can cause an adult child caring for a parent with dementia to become emotionally disengaged. Being emotionally disengaged is evidence of compassion fatigue.

## 5. Consequences of Compassion Fatigue

Compassion fatigue has negative outcomes for formal caregivers such as depression, isolation; physical symptoms of insomnia, fatigue, or weight changes; increased errors at work and job dissatisfaction [1–5, 7, 20, 21, 29, 36]. While the likely consequences of compassion fatigue in informal caregivers have not been explored in research published in the current literature, we propose that compassion fatigue may lead to negative outcomes for both the caregiver and the care recipient. Drawing upon formal caregivers' negative outcomes, and because similar concepts, such as depression and burden, have negative outcomes for informal caregivers, we propose that informal caregivers for family members with dementia who develop compassion fatigue may suffer depression, burden, caregiver strain, and a decreased relationship quality with the care recipient. Compassion fatigue may also determine whether or not the caregiver continues to care for their family member with dementia or if the person with dementia is placed in an institutional setting or cared for by another person.

### 5.1. Depression

Informal caregivers for people with dementia are already at risk for depression as found in multiple studies [8, 14, 15, 48, 49]. Yaffe et al. [15], in a large, multi-site study of 5,788 patients, found that caregivers with at least six depressive symptoms were 1.18 more times likely to place the care recipient in a nursing home than a caregiver with five or less depressive symptoms. Research suggests a high level of depression among dementia caregivers finding mean score of 21.47 [50] on the Center for Epidemiologic Studies Depression Scale (CES-D) [51] where higher scores are indicative of greater depressive symptomatology and a score of 16 indicates depression [51].

### 5.2. Burden

Caregiver burden is related to competing life demands, and the more competing life demands, the greater the opportunity for both objective and subjective burden. As discussed, dementia caregivers experience burden [37, 49, 52–57] and compassion fatigue could lead to even greater burden; it is also possible that the relationship between burden and compassion fatigue is reciprocal. Further, caregivers for family members with dementia experience burden even when the family member resides in an institution [58]. A study of 172 caregivers found that caregivers had no significant difference in level of burden related to care recipient residence [58]. This study also found a relationship between level of education and income to caregiver burden; less educated and lower income caregivers were at greater risk for burden [58]. Caregiver depression is a major factor related to burden, and Papastavrou and colleagues [58] found that 85 caregivers (49.41%) had scores above the risk level for development of clinical depression. Caregivers for family members with dementia often experience burden, which may be coupled with depression. This combination of depression and burden places the care recipient at risk for negative consequences as well.

### 5.3. Caregiver Strain

Relationship quality is closely associated with caregiver role strain [26]. Caregivers with positive relationship quality have less caregiver strain because they are able to find meaning in caregiving and, correspondingly, caregivers with weak relationship quality will experience greater caregiver strain [26]. High mutuality in family caregivers is a protective factor for negative caregiving outcomes, such as depression, burden, and resentment [25–27, 59–61].

### 5.4. Decreased Relationship Quality

Relationship quality is different for all family caregiving dyads; however, we propose that those caregivers affected by compassion fatigue are most likely to have a decreased sense of relationship quality with their family member. In a qualitative study of 11 daughter caregivers, the authors described four of the caregivers as using a dispassionate approach [62]. The daughters using the dispassionate approach demonstrated decreased or only superficial communication, defined their caregiving role in terms of the tasks, and gave little thought to the future [62]. The dispassionate approach reported by the researchers presumably demonstrates a decreased relationship quality between caregiver and care recipient. Mutuality, a positive relationship quality [26], is affected by the caregiving experience. In a study of 87 caregivers for family members with cancer, the researchers found that difficult caregiving situations arose from a poor relationship [63].

### 5.5. Termination of Caregiving, Abuse, and Neglect

Family members feel guilt when they are not able to care for their loved ones at home [64, 65]. When examining Korean family caregivers, Park et al. [66] found more than half stated they felt guilty about deciding to place their loved one in an institution. One daughter recollected, “I couldn't sleep for days. Thoughts like why did I do such a thing? Or what would her life be like? Would not let me go to sleep” [66], and another daughter recalled, “Sometimes I wonder how much she must hate me inside for doing this to her. I feel guilty because I feel she's saying “How could you abandon me here?” [66]. Family members who need to make this decision often have a difficult time and experience what Park et al. describe as deep sorrow [66].

Another study exploring nursing home placement by wife and daughter dementia caregivers found an interaction between kinship and role captivity, role overload, and day care use [67]. Role captivity, described as how trapped and constrained the caregiver feels, and role overload, described as overwhelming caregiving demands, were described by the authors to be primary subjective stressors in nursing home placement. This study of 371 caregivers revealed that regardless of relationship, those caregivers with high role captivity placed their family member in nursing homes earlier than caregivers who had low role captivity, while only wives placed their husbands in nursing homes earlier when role overload was high [67]. This suggests that caregivers who feel trapped and constrained, like caregivers with compassion fatigue who suffer hopelessness and helplessness, are more likely to place a family member in a nursing home earlier than those who do not feel this way [67].

Caregiver depression and burden place care recipients at risk physically and emotionally. Thorpe et al. [68] examined caregiver psychological distress in 1,406 dementia caregivers and found caregiver distress to be significantly negatively related to a care recipient receiving an influenza vaccination. Gainey and Payne [55] conducted a review of 751 elder abuse cases from Adult Protective Services and found that caregiver burden did not correlate with increased abuse but was associated with increased neglect and exploitation. 

People with dementia are not only at risk for neglect but also at increased risk for physical abuse from the caregiver. In a sample of 417 informal caregivers, Shaffer et al. [69] discovered that as a caregiver feels increasingly resentful towards the care recipient, he or she is more likely to abuse the care recipient. Pérez-Rojo et al., in a sample of 45 Spanish caregivers from Madrid, demonstrated that the greater a dementia care recipient is dependent upon the caregiver, the less help a caregiver receives and the more aggressive the dementia patient's behaviors, the more likely the caregiver is to become aggressive and possibly abusive [70]. Pérez-Rojo et al.'s study did not compare Spanish caregivers to other ethnic groups and thus more research is needed in a more diverse sample. The studies presenting the negative consequences of caregiving generally examined large populations and multiple studies found the same results. While no studies specifically examined the negative effects of compassion fatigue on informal caregivers and care recipients, this is not unexpected because the concept has not been previously defined for this population.

## 6. Conclusions

Compassion fatigue in family caregivers has not been the focus of a primary research study. In this analysis, we have demonstrated that the concept applies to family caregivers. We propose that compassion fatigue in family caregivers may be the combination of hopelessness, helplessness, apathy, and emotional disengagement that occurs after a prolonged exposure to suffering. Compassion fatigue depends upon concern and an empathic response from the caregiver for the care recipient with dementia, and this concern and emotional attachment is the motivation for the caregiver to relieve the suffering. If a caregiver experiences an empathic response, coupled with competing life demands and a lack of satisfaction from caregiving, they may be at risk for compassion fatigue.

Compassion fatigue is a process; it is the end result of a cascade of events that in turn may lead to caregiver depression, increased burden, caregiver strain, and decreased relationship quality. The consequences of compassion fatigue may also lead to termination of the caregiving relationship through premature admission to a long-term care facility or relinquishing care to another family member or to behaviors of abuse or neglect of the care recipient. Research has shown that caregivers of family members with dementia experience the events that lead formal caregivers towards compassion fatigue: empathic ability and concern, prolonged exposure to perceived suffering, no sense of satisfaction, and competing life demands. Likewise, family caregivers exhibit the likely indicators of compassion fatigue and also endure depression and burden.

By applying the concept of compassion fatigue to family caregivers, we have an enhanced understanding of the caregiving experience. This application suggests the following research questions.

- How does compassion fatigue present in caregivers of a family member with dementia?
- To what extent do caregivers for a family member with dementia experience compassion fatigue? Are the compassion fatigue process and its antecedents and outcomes similar to those of formal caregivers (prior research)?
- How is the nature of the relationship between caregiver and care recipient related to the caregiver's risk for compassion fatigue?
- What instruments are available to measure compassion fatigue in family caregivers, and do these measures demonstrate reliability and validity?
- How can nurses intervene to decrease a caregiver's risk for, or experience of, compassion fatigue and its consequences?

It is important for nurse researchers to investigate this concept to fully understand compassion fatigue and the consequences it may have on the caregiving dyad. If health care professionals know the specific cascade of events leading to compassion fatigue in the family caregiving population, they may intervene and interrupt this cascade at many points. If the cascade is not interrupted and the caregiver develops compassion fatigue, there may be risk for termination of the caregiving relationship, abuse, or neglect [13, 14]. It is possible that compassion fatigue is analogous to a syndrome that could include this myriad of indicators and consequences as symptoms of an overarching construct. Future research could explore this possibility.

Knowledge of compassion fatigue in family caregivers will lead to development of interventions to reduce this devastating outcome. Interventions may include anticipatory guidance for caregivers identified at risk for compassion fatigue. Nurses are also able to reduce perceived care recipient suffering through interventions such as medication and behavioral management. Additional interventions may utilize online resources, such as chat groups, as well as phone interventions that fit into the complex lives of caregivers.

## Figures and Tables

**Table 1:** Definitions of concepts and key terms.

Authors, year	Population	Purpose	Definition of concepts and key terms
Joinson, 1992	Nurses	Description of compassion fatigue, how to recognize it, and how to prevent it	*Compassion fatigue*: unique form of burnout linked to caregiving professionals, can be emotionally devastating, causes loss of ability to cope, anger, apathy, depression, and ineffectiveness

Figley, 2002	Psychotherapists	To discuss compassion fatigue as experienced by psychotherapists and contrast the concept with burnout and countertransference	*Secondary traumatic stress*: the natural behaviors and emotions that arise from caring for someone suffering from a traumatizing event
			*Compassion fatigue*: tension and anxiety that occurs from reexperiencing traumatic events with the patient, sense of helplessness and confusion, isolation from support
			*Burnout*: physical, mental, and emotional exhaustion that occurs from continuous emotionally demanding encounters

Keidel, 2002	Hospice caregivers	To discuss how burnout affects hospice caregivers and to examine causes of stress	*Burnout*: “syndrome of physical exhaustion including a negative self-concept, negative job attitude, and loss of concern and feeling for patients”
			*Compassion fatigue*: form of burnout affecting people in caregiving professions, less abrasive term than burnout

Huggard, 2003	Physicians	To shed light on the subject of compassion fatigue in medical education programs	*Empathy*: validating the client's world through understanding the “story behind the story”
			*Compassion fatigue*: based on Figley's definition, a sudden stress response with symptoms disconnected from the real cause and being empathic places someone at risk

McHolm, 2006	Nurses	To use God and scriptures to examine what can be done for nurses experiencing compassion fatigue and to see how compassion fatigue is different than burnout	*Compassion*: being aware of the suffering of another and the strong desire to alleviate the suffering
			*Compassion fatigue*: the emotional, social, and spiritual exhaustion that causes a decline in the desire, ability, and energy to feel and care for others, lost ability to experience satisfaction and joy in profession and personal life
			*Burnout*: becoming less empathic to patients and displaying negative behaviors to coworkers, “candle that goes out because the wax has been used up”

Sabo, 2006	Nurses	To examine the effects that caring has on nurses' health	*Compassion*: “experience of feeling with another while recognizing that the feelings of one are not the same as another”
			*Empathy*: awareness of a patient's feelings, sharing this with the patient, and the patient's awareness that the nurse feels this
			*Compassion fatigue*: the acute onset of a combination of secondary traumatic stress and burnout
			*Burnout*: a gradual negative change in professional attitude to job strain

Schulz, et al., 2007	Family caregivers	To discuss the relationship between patient suffering and caregiver compassion	*Suffering*: bearing or undergoing of pain, distress, or tribulation (Oxford English Dictionary, 1989)
			*Compassion*: sense of shared suffering accompanied by a desire to relieve the suffering
			*Compassion fatigue*: the stress, strain, and wariness that arises from caring for a person suffering from a medical or psychological problem

Adams et al., 2008	Social workers	To investigate the differences between secondary traumatic stress and job burnout and to see if secondary trauma can predict psychological distress	*Compassion fatigue*: a formal caregiver's inability or disinterest in being empathic or sharing the suffering of clients
			*Burnout*: the emotional exhaustion, depersonalization, and reduced personal accomplishment that arises from prolonged exposure to demanding interpersonal relationship and stressful environments

Marr, 2009	Physicians	A personal reflection	*Compassion*: the suffering with the sufferer that occurs in the moment because of the relationship between beings; what we do, not who we are

Robins et al., 2009	Physicians, nurses, mental health practitioners, allied health practitioners	To examine the effect providing care has on health care workers and trauma workers, and to examine the relationship between secondary traumatic stress, empathy, spirituality, and coping	*Compassion fatigue*: the symptoms and emotional responses the occur from caring for traumatized persons, same as secondary traumatic stress and vicarious traumatization

Ward-Griffin et al., 2011	Nurse-daughters caring for elderly parents	To examine compassion fatigue in nurse-daughter caregivers and identify the effect of the environment on compassion fatigue	*Compassion fatigue*: distinct from burnout, a condition affecting physical, emotional, and social health and well-being, “living on the edge” where expectations exceed resources
